# Plasticity of lung development in the amphibian, *Xenopus laevis*

**DOI:** 10.1242/bio.20133772

**Published:** 2013-10-16

**Authors:** Christopher S. Rose, Brandon James

**Affiliations:** James Madison University, Department of Biology, Biosciences 2028, Harrisonburg, VA 22807, USA

**Keywords:** Lung, Plasticity, Respiration, Amphibian

## Abstract

Contrary to previous studies, we found that *Xenopus laevis* tadpoles raised in normoxic water without access to air can routinely complete metamorphosis with lungs that are either severely stunted and uninflated or absent altogether. This is the first demonstration that lung development in a tetrapod can be inhibited by environmental factors and that a tetrapod that relies significantly on lung respiration under unstressed conditions can be raised to forego this function without adverse effects. This study compared lung development in untreated, air-deprived (AD) and air-restored (AR) tadpoles and frogs using whole mounts, histology, BrdU labeling of cell division and antibody staining of smooth muscle actin. We also examined the relationship of swimming and breathing behaviors to lung recovery in AR animals. Inhibition and recovery of lung development occurred at the stage of lung inflation. Lung recovery in AR tadpoles occurred at a predictable and rapid rate and correlated with changes in swimming and breathing behavior. It thus presents a new experimental model for investigating the role of mechanical forces in lung development. Lung recovery in AR frogs was unpredictable and did not correlate with behavioral changes. Its low frequency of occurrence could be attributed to developmental, physical and behavioral changes, the effects of which increase with size and age. Plasticity of lung inflation at tadpole stages and loss of plasticity at postmetamorphic stages offer new insights into the role of developmental plasticity in amphibian lung loss and life history evolution.

## Introduction

Amphibians use many different organs for gas exchange throughout their lives. These include lungs, internal and external gills, the internal skin of the mouth and pharynx (buccopharyngeal epithelium), and the external skin, which can be specialized into respiratory papillae, folds, and expanded tail fins (Duellman and Trueb, 1986; Noble, 1931). Skin can function in respiration in both water and air and can thus be used by all but the most terrestrial amphibians throughout life. In contrast, gills function only in water and lungs only in air so their use in amphibian respiration varies significantly with life history and ecology. Interestingly, frogs delay lung inflation (meaning the initial act of inflating their lung rudiments with air) to different stages after hatching. Whereas the permanently aquatic pipid, *Xenopus laevis*, starts using lungs soon after hatching, the American bullfrog *Rana catesbeiana* (*Lithobates catesbeianus*) and other ranids do not inflate theirs until mid-tadpole stages, and bufonids, megophryine pelobatids and *Scaphiopus* delay inflation until metamorphosis (Burggren and Just, 1992; Ultsch et al., 1999; Wassersug and Seibert, 1975). This variation raises two questions about tetrapod lungs that can only be addressed in amphibians: when does lung respiration become obligatory and how plastic is lung development?

Although tadpoles with lungs that live in normoxic water (meaning water that is 80–100% saturated with dissolved oxygen) usually breathe air, lung respiration is generally not considered essential for tadpole survival (Burggren and Just, 1992; Pronych and Wassersug, 1994; Ultsch et al., 1999). *Xenopus* tadpoles obtain 17% of their oxygen from air (Feder and Wassersug, 1984) and *Rana catesbeiana* tadpoles change from 15% at the start of lung use to 80% at the end of climax metamorphosis; lungs are considerably less involved in CO_2_ removal than oxygen uptake (Burggren and West, 1982). Lung inflation contributes positive buoyancy, which facilitates locomotion and feeding in still or slow water, but at the cost of swimming downwards to maintain position in the water column (Ultsch et al., 1999). Lung respiration also allows the buccopharyngeal surfaces of suspension feeding forms like *Xenopus* to be more fully committed to feeding (Feder et al., 1984; Wassersug and Murphy, 1987). Lung respiration becomes most crucial in hypoxic water since unlike gills and skin, lungs do not pose the risk of oxygen loss to the water (Feder and Wassersug, 1984). *Xenopus* and *Rana* tadpoles respond to acute hypoxia by increasing the frequency of breathing, starting at the earliest stages of lung use (Feder, 1983; Feder et al., 1984; Pan and Burggren, 2010; West and Burggren, 1982). Whether tadpoles can use lung respiration to survive chronic hypoxia remains to be seen (Ultsch et al., 1999).

Amphibian larvae have been shown to exhibit plasticity in lung development in response to several conditions. *Rana catesbeiana* tadpoles raised in hypoxic water developed oversized lungs (Burggren and Mwalukoma, 1983; Burggren and Just, 1992) and *Discoglossus pictus* tadpoles raised in hyperoxic water developed undersized lungs (Barja de Quiroga et al., 1989). These results suggest that lung development and growth are directly affected by the availability of oxygen. Being deprived access to air caused *Xenopus* tadpoles (Pronych and Wassersug, 1994) and *Ambystoma maculatum* larvae (Bruce et al., 1994) to develop half-sized lungs, which suggests that lung development also relies upon the physical forces exerted during inflation by air. That lung loss has evolved at least twice in salamanders (Dunn, 1923; Dunn, 1926) and once in each of caecilians and frogs (Bickford et al., 2008; Hutchison, 2008; Nussbaum and Wilkinson, 1995) raises the possibility that plasticity in lung development might allow some normally lunged amphibians to survive without inflating or even developing lungs.

In the one previous experiment on long-term air deprivation in frogs, the effects of air deprivation on developmental rate, survivorship and heart development and function were severe enough for the authors to conclude that lung respiration was obligatory in *Xenopus laevis* tadpoles (Pronych and Wassersug, 1994; Wassersug, 1996). We repeated this experiment to identify the specific stage at which lung respiration became obligatory. When raising *Xenopus laevis* in well circulated, normoxic water without access to air from embryonic stages before the onset of breathing, we found that air-deprived (AD) tadpoles can routinely complete climax metamorphosis. Further, these animals appear fully viable with lungs that are either severely stunted and uninflated or absent altogether and will survive, grow and mature without breathing air for at least two years.

This is the first demonstration that lung development in a tetrapod can be inhibited by environmental factors and that a tetrapod that relies significantly on lung respiration under unstressed conditions (Boutilier, 1984; Boutilier et al., 1986; Willem, 1931) can be raised to forego this requirement without adverse effects. This study uses whole mounts and histology to compare lung development in untreated and AD animals and to investigate the ability of air-restored (AR) animals to recover lungs as a function of developmental stage, age and time after air restoration. This study also examines the relationship of swimming and breathing behaviors to lung recovery in tadpoles and frogs.

## Results

### Lung development and onset of breathing in untreated animals

**Fig. 1.**
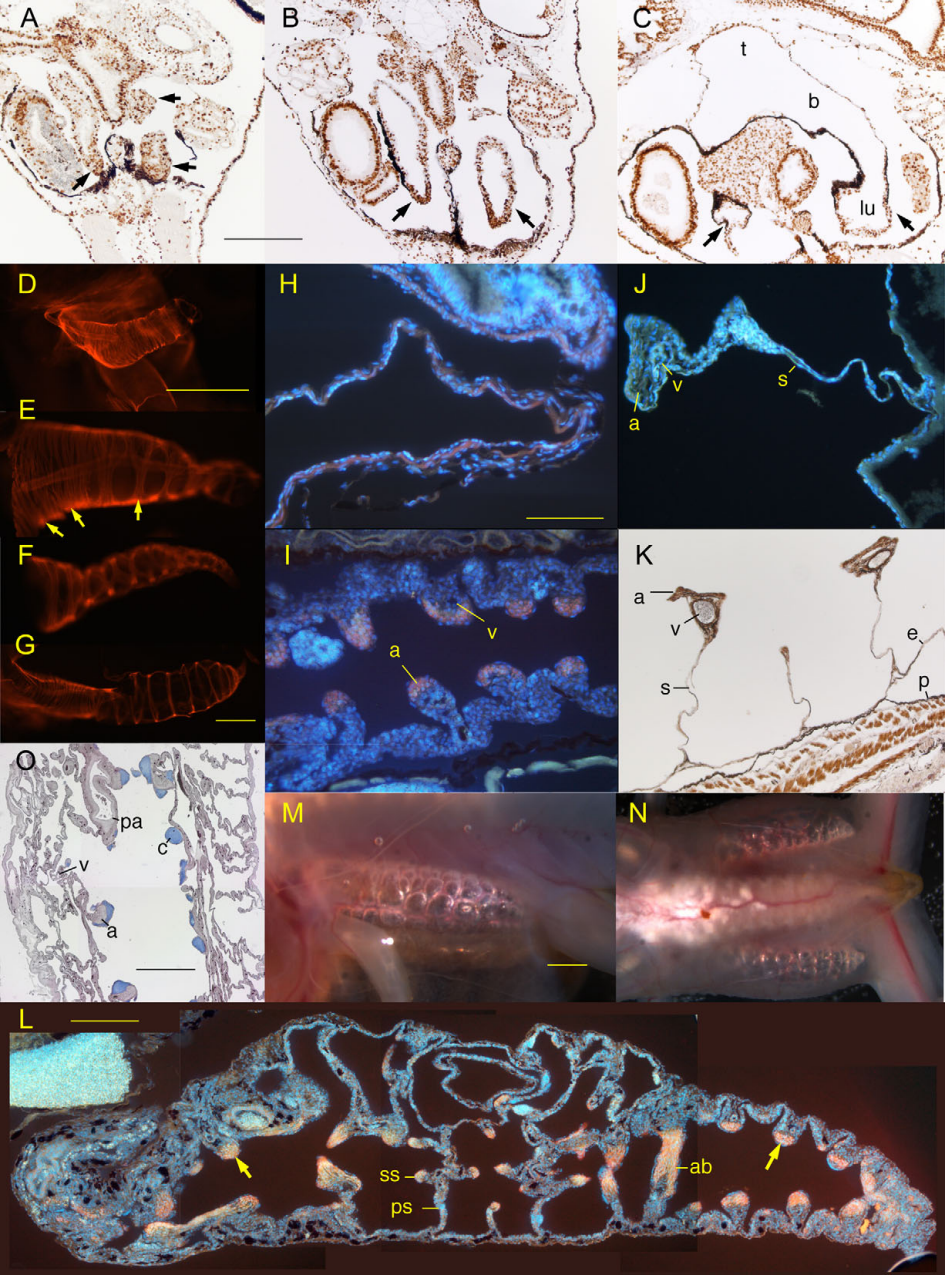
Lung development in untreated *Xenopus*. (A–C) Frontal sections (anterior is up) showing lungs (arrows) at (A) NF 46 just before first breath (fb), (B) NF 47 three days after fb showing expansion of lumen, and (C) NF 47 six days after fb showing thinning of walls of trachea (t), bronchi (b) and lungs (lu) except at the posterior tips; brown are BrdU-labeled nuclei. Lungs show similar levels of BrdU labeling as other tissues before and after inflation. (D–G) Whole mounted lungs (anterior is left) with fluorescently stained smooth muscle actin at (D) NF 47 showing closely spaced bands throughout the length, (E) NF 52 showing difference in spacing and branching of actin bands anteriorly (left arrows) and posteriorly (right arrow) and pulmonary artery, (F) NF 52 with an uninflated tip, and (G) NF 52 with full inflation showing closely spaced bands anteriorly (left) and widely spaced bundles posteriorly (right). (H–J) Frontal sections (anterior is left) with fluorescently stained smooth muscle actin (red) and nuclei (white) at (H) NF 54 showing a continuous layer of actin, (I) NF 54 showing thickening of the lung wall and the appearance of epithelial folds with actin bundles (a) in the crest and rudimentary veins (v) in the base, and (J) NF 58 showing a primary alveolar septum (s) comprised of two epithelial sheets and an expanded crest containing an actin bundle (a) and vein (v). (K) Frontal section of more advanced septa at NF 58 showing flattening and extension of the actin layer (a, brown) and well developed veins (v), lung epithelium (e) and peritoneum (p). (L) Frontal section through the peripheral space of the lung (anterior is left) with fluorescently stained smooth muscle actin (red) and nuclei (white) at NF 63 showing primary (ps) and secondary alveolar septa (ss) in the middle regions, epithelial folds (arrows) in the anterior and posterior regions, and an actin-rich band (ab) posteriorly. (M,N) Lateral views (anterior is left) of live albinos at (M) NF 58 showing lateral subdivision of the smooth muscle-bound compartments and (N) NF 66 showing lungs extending the full length of the pleuroperitoneal cavity. (O) Frontal section (anterior is up) through an adult lung showing the undivided central space, partitioned peripheral spaces and cartilages (c, blue), smooth muscle bundles (a), pulmonary artery (pa) and veins (v) lining the border between these spaces. Scale bars are 0.5 mm for A–C and H–L, and 1 mm for D–G, M–O.

To assess the effects of air deprivation and air restoration, it is first necessary to describe normal lung development during the stages affected by these treatments. Early NF 46 marks the onset of breathing as determined by the onset of spontaneous swimming towards the surface, quick departures from the surface followed by the release of bubbles, and the switch from sinking to floating when specimens are put in fixative. At NF 46 before the first breath (Fig. 1A), the trachea and bronchi are well formed, the lungs extend most or all the length of the pleuroperitoneal cavity, and the ventral surfaces of the trachea, bronchi, lungs and esophagus are overlain by a strongly pigmented peritoneum (this term is preferred here to pleura since this membrane continues as the lining of the pleuroperitoneal cavity and does not yet lie close to ventral lung surfaces or cover dorsal lung surfaces). Lung inflation appears to begin during the first six days after the onset of breathing (NF 46–47) and is marked by dramatic expansions of the lumens of trachea, bronchi and lungs and thinning of anterior lung walls into thin membranes with flattened cells (Fig. 1B,C); lung walls in the posterior tip remain several cells thick at NF 47–49 (11–17 days pf). Lung inflation is not accompanied by a noticeable increase in BrdU labeling in lung tissue (Fig. 1A–C). At NF 50, each bronchus acquires a pouch (bronchial diverticulum) that extends dorsally to abut the oval window of the ear capsule and back muscles.

Waterman's description of lung differentiation and the origin of alveolar septa in *Rana (Lithobates) pipiens* (Waterman, 1939) provides a standard for understanding this process in *Xenopus*. According to Waterman, the cuboidal epithelium of the early lung rudiment separates into an inner epithelium, outer pleura, and scattered strands of connective tissue in between, all with flattened cells (Waterman, 1939). In the space between epithelium and pleura appear first a deep layer of smooth muscle cells and later a more superficial “lattice-like” plexus of veins. The epithelium expands outwards into the spaces between veins, and sheets of epithelium and connective tissue on opposite sides of a vein gradually come together to internalize the vein and form an internal partition or primary alveolar septum. As this happens, the layer of smooth muscle disappears in the spaces between the veins and thickens into bundles that lie deep to the plexus of veins. These events occur between late embryonic and late tadpole stages in *Rana pipiens*; judging from figure 3 of Waterman, lung inflation occurs after the smooth muscle layer appears but before veins appear (Waterman, 1939).

*Xenopus* generally follows the same sequence of events, but unlike *Rana*, its lung wall does not separate into thin membranes with flattened cells until after inflation and its lattice of smooth muscle bundles never resembles the orthogonal pattern figured by Waterman. As the timing of events can vary with stage and lung region in *Xenopus*, earliest stages of occurrence are reported here. By NF 47 (the earliest stage used for actin staining in whole mounts), the smooth muscle in the lung wall is arranged in bands of densely staining fibers (Fig. 1D, though actin can appear as a continuous layer in sectioned material until NF 54, Fig. 1H). The muscle bands and the fibers comprising them are aligned transversely, i.e., perpendicular to the long axis of the lung cylinder and to the pulmonary artery, which lies deep to the muscle bands in the anterior three quarters of the lung. By NF 52, anteriorly, the lung is cylindrical, the muscle bands are thin, parallel to each other and closely spaced, and adjacent ones are connected at junctions where one thick bundle splits into two thinner ones (Fig. 1E). Posteriorly, the lung tapers to a tip, the muscle bands are widely spaced and thicker (and now more aptly described as bundles), and the two thinner bundles leaving a junction are aligned obliquely to the lung axis (Fig. 1E). In the spaces between bundles, the lung wall is composed of a thin, almost transparent epithelium that bulges outwardly. The lung can have a sharp taper at its posterior tip or be wider throughout the posterior half than the anterior half (Fig. 1F,G). The shape becomes more uniform at later stages, with a gentle taper in its posterior half or third.

At NF 54–57, muscle bundles that are widely separated begin to shift inward relative to the lung surface. At NF 54, the muscle bundles are situated in the crests of inwardly directed epithelial folds, the bases of which show the rudiments of veins (Fig. 1I). By NF 57, these folds have become dramatically longer and thinner to form the primary alveolar septa (Fig. 1J,K). The crests of the septa exhibit a thick muscle bundle and differentiated vein. By NF 58, the crests of primary septa have formed bridges with adjacent septa, and the resulting three-dimensional network supports thick veins and bands of smooth muscle that extend into the interior of the lung (Fig. 1L). Visualized in albino whole mounts, the muscle bundles, which previously divided the lung into disk-like compartments along its length, now subdivide each compartment into 3–4 smaller globular compartments across its width (Fig. 1M,N). By NF 63, the primary septa are thicker and more folded and interconnected, and some exhibit secondary septa forming on their sides (Fig. 1L). The lung is separated into an open central space and a highly compartmentalized peripheral space, the inner surfaces of which are lined with smooth muscle (Fig. 1O). By NF 65, a network of hyaline cartilage bars has begun to chondrify in the walls of the bronchus and in the septa lining the central space (Fig. 1O and figure 9 of Konno et al., 2013). Staging the appearance of specific cell types (e.g., ciliated epithelial cells, endothelial cells, pneumocytes, and lymphatic cells) and other extracellular matrix components (e.g., collagen and elastic fibers) is beyond the scope of this paper.

Of the 74 lungs scored in dissected tadpoles, 74% were longer than three quarters the length of the pleuroperitoneal cavity (i.e., greater than 0.75 PCL) and the rest were 0.5–0.75 PCL; the shorter lungs were in specimens ≤NF 55. Of the 58 lungs scored in dissected frogs, 86% were 0.75–1 PCL and the rest were 0.5–0.75 PCL; the shorter lungs were in recently metamorphosed specimens that had yet to accumulate fat bodies. Of the 10, 26 and 34 lungs observed through the skin of albino tadpoles, metamorphs and frogs, 90, 88 and 74% respectively were 0.75–1 PCL and the rest were 0.5–0.75 PCL (Fig. 1L,M). On the basis of these observations, normal sized lungs are reached between NF 46 and 49 and are 0.5–1 PCL, inflated, straight and not filled with tissue.

### Lung development in AD animals

**Fig. 2.**
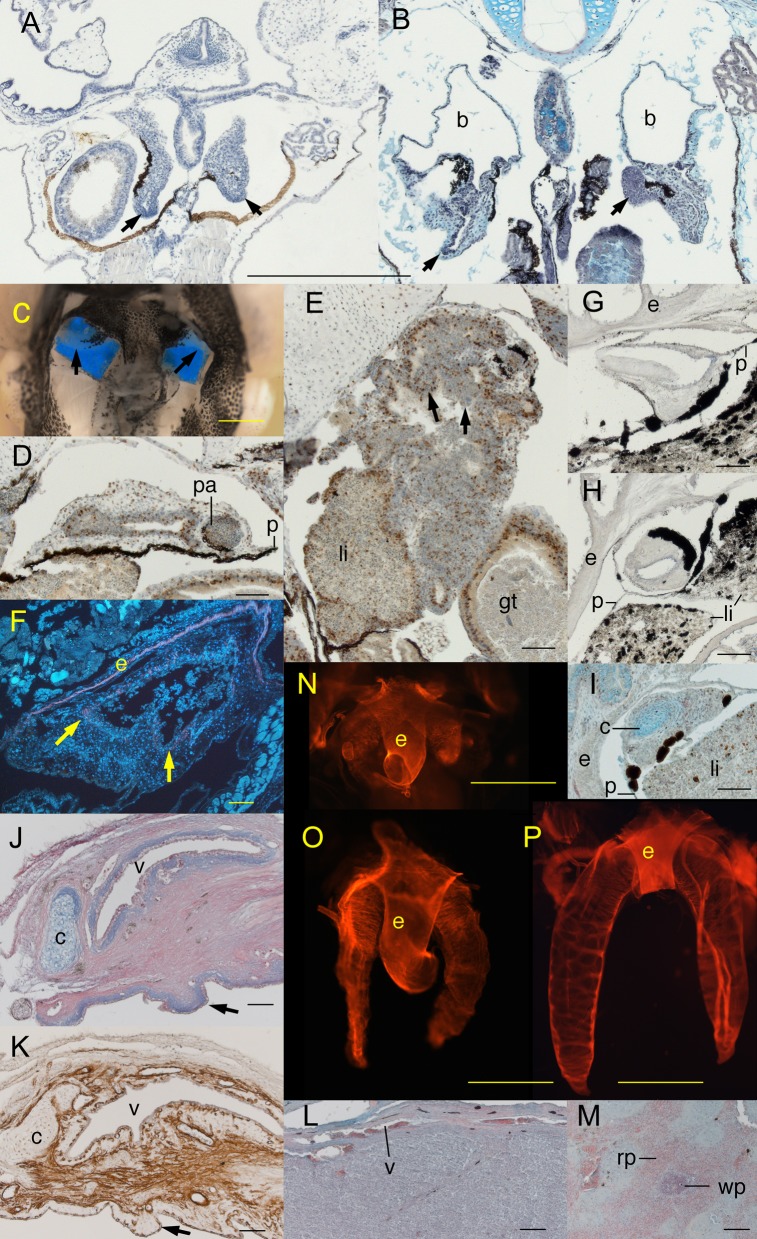
Lung development in air-deprived (AD) and air-restored (AR) *Xenopus*. (A,B) Frontal sections of AD tadpoles at (A) NF 46 showing normal lung development and (B) NF 49 showing expanded bronchi (b) and compressed, folded lungs (arrows). (C) Ventral view of a dissected AD NF 57 tadpole showing translucent stunted lungs against a blue background. (D,E) Frontal sections of an AD NF 57 tadpole showing (D) ventrally a compressed, thick walled bronchus with a pulmonary artery (pa) and small lumen anterior of the peritoneum (p) and (E) dorsally a compressed, folded lung abutting the liver (li) and gut tube (gt); arrows indicate incipient epithelial folds and brown is BrdU labeling. (F) Frontal section of a stunted AD NF 57 lung with fluorescently stained smooth muscle actin (red) and nuclei (white) showing little actin adjacent to the epithelium (arrows) and partial filling of the lumen by blood cells (the continuous actin layer is in the wall of the esophagus, e). (G–I) Frontal sections of AD NF 66+ frogs showing (G) ventrally a compressed bronchus, (H) dorsally the absence of a lung and the bronchus extending into the peritoneum-lined space normally occupied by the bronchial diverticulum, and (I) cartilage (c, blue) and BrdU labeling (brown) in the wall of a compressed bronchus. (J,K) Sagittal sections through the wall of a 19-month old AD lung showing a thick connective tissue layer containing cartilage (c), veins, and bands of actin fibers (pink in J and brown in K), and large incipient epithelial folds (arrows) protruding into the lumen (lower side). (L) A tissue-filled lung showing a thin wall, vein (v) and lumen filled with blood cells. (M) A section through the spleen of an adult frog showing the red (rp) and white pulp (wp). (N–P) Dorsal views of whole-mounted actin-stained lungs and esophagus (e) of (N) an AD NF 51 tadpole with weakly defined, closely spaced actin bands, (O) an AR NF 53 tadpole 9 days after air restoration with actin bundles more closely spaced than in untreated NF 52 lungs, and (P) an AR NF 59 tadpole 4 days after with widely spaced actin bundles but not the lateral subdivisions seen in untreated NF 59 lungs. Anterior is up for frontal views A–I and left for sagittal sections J,K. Scale bars are 0.5 mm for A,B; 1 mm for C, N and O; 0.1 mm for D–M; and 2 mm for P.

AD lungs at NF 46 were cylindrical and of normal length (0.5–1.0 PCL), and most had thick walls and no sign of a lumen (Fig. 2A). Two of four specimens that became AD after starting to breathe air had lumens but only one lumen was wider than the lung wall. Whereas the eight animals that became AD before starting to breathe all survived the transfer, the four that became AD after breathing died less than 12 hours after the transfer.

AD lungs at NF 49–51 were 1.5–2 times longer than at NF 46, but at 0.25–0.5 PCL were shorter than normal lungs (Fig. 2B). They were compressed into irregular shapes with their posterior tips folded back against the anterior wall of the pleuroperitoneal cavity and their walls enclosing portions of the pigmented peritoneum. Lung walls were multiple cells thick and lumens were narrow and discontinuous. The smooth muscle in AD lungs at NF 51–54 was weakly staining and bundles lacked the sharp edges, uniform spacing and orderly arrangement of bundles in untreated specimens (Fig. 2N).

In AD specimens at NF 56–57 (Fig. 2C), the bronchi were compressed against the sides of the esophagus, remained anterior of the peritoneum, and extended dorsally into the spaces normally occupied by the bronchial diverticula; their dorsal tips were not expanded and usually abutted trunk muscle and/or ear capsule (Fig. 2D). Lungs, if present, were usually highly infolded, had little or no lumen and abutted against liver, fat bodies or intestines (Fig. 2E), though in one specimen they extended freely into the pleuroperitoneal cavity and exhibited a lumen wider than the lung walls. Some lumens were partially filled with what appeared to be blood cells (Fig. 2F). Lung walls were comprised of a thin epithelium and pleura and a thick connective tissue layer containing loose cells, small amounts of disorganized actin fibers close to the epithelium (Fig. 2F), veins and occasionally pulmonary arteries (Fig. 2D,E). Epithelial folds indicating incipient septa were evident in both the freely extended and infolded lungs (Fig. 2E). The one BrdU-treated specimen did not exhibit conspicuous labeling in lung tissue (Fig. 2D,E).

One AD frog at two months post NF 66 exhibited small, pigmented cylinders of undifferentiated tissue with continuous lumens in place of bronchi and bronchial diverticula and no lung tissue inside the cavity (Fig. 2G–I). The lung of a 19-month old AD frog that was 0.25 PCL and extended into the cavity exhibited a thick connective tissue layer with veins and cartilages, disorganized smooth muscle bundles close to the epithelium and parallel smooth muscle bundles close to the pleura (Fig. 2J,K). There was no sign that septa formation had progressed beyond the stage of epithelial folds.

Of the 225 lungs scored in AD specimens (Table 1), none showed any evidence of inflation, 17% (all in whole mounts) appeared to be absent altogether, 74% were 0.1–0.25 PCL (Fig. 2H), and 10% were 0.25–0.5 PCL. Of the 20 lungs that were 0.25–0.5 PCL, 15 belonged to tadpoles less than NF 55. Of the 112 specimens with two scoreable lungs, 72% had two straight lungs, 15% a curled left lung and straight right one, 10% two curled lungs, and 3% a curled right one and straight left one. Of the 14 specimens with stunted lungs of noticeably different lengths, 64% had longer left lungs.

**Table 1.**
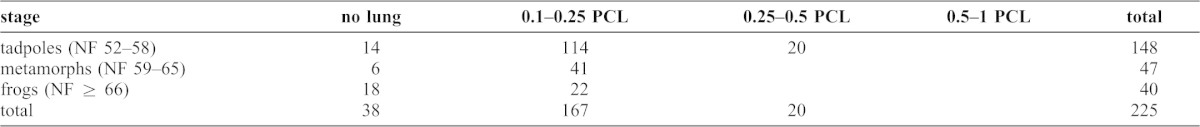
Lung length in scored AD animals as a function of developmental stage.

### Lung development in AR animals

AR lungs were either stunted and uninflated or showed some degree of recovery to normal lung size and inflation. Stunted AR lungs resembled AD lungs of the same stages in shape, size, position, lack of conspicuous BrdU labeling, and the development of lumens, blood vessels, cartilages, smooth muscle and epithelial folds (Fig. 2). Recovered AR lungs resembled the inflated lungs of untreated specimens, although the formation of primary septa appeared to lag behind that of equivalently staged untreated tadpoles (Fig. 2N–P).

Three tissue-filled lungs were found in two frogs that had been returned to air for 1 and 30 days. The former specimen had two tissue-filled lungs of unequal length and the latter appeared to lack one lung entirely. Given the short period of air restoration for the former specimen and the occurrence of partially filled AD lungs (Fig. 2F), it is likely that tissue filling happened during air deprivation and not as a result of air restoration. Histologically, the lung walls resembled those of other stunted AR and AD lungs at postmetamorphic stages (Fig. 2L). However, the lumens were partially or completely filled with what appeared to be blood cells, which included few if any erythrocytes and resembled the white pulp of the spleen (Fig. 2M) (see also figures in Konno et al., 2013; Wiechmann and Wirsig-Wiechmann, 2003).

AR tadpoles at NF 55–58 tended to recover lungs of normal length (Table 2). Recovery started the day after being restored to air and was complete by day 8. There were no obvious differences in probability, rate or extent of recovery among the four tadpole stages that were tested. In contrast, lung recovery in AR metamorphs and frogs was slower and less likely to occur (Tables 3, 4). Of the 22 lungs in frogs that were AR for 4–8 weeks, 59% were stunted to the point of appearing absent. However, one of the 10 frogs that were returned to air for one day had lungs that were 0.5 and 0.75 PCL, which suggests that lung recovery can still occur in frogs and as quickly as in tadpoles.

**Table 2.**
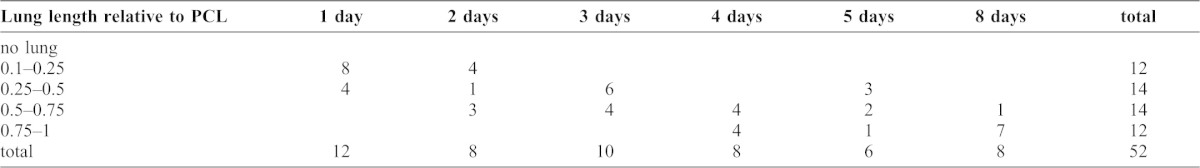
Lung length in scored AR tadpoles as a function of time after air restoration.

**Table 3.**
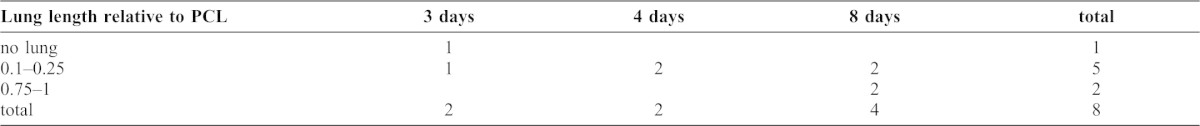
Lung length in scored AR metamorphs as a function of time after air restoration.

**Table 4.**
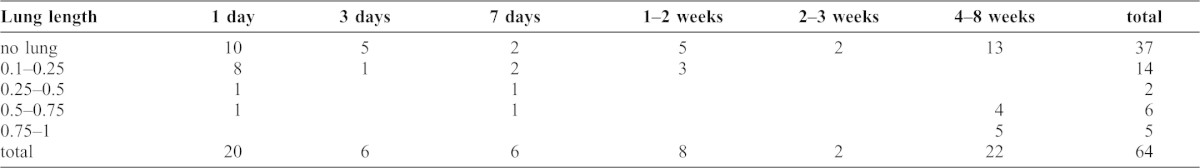
Lung length in scored AR frogs as a function of time after air restoration.

Of the 128 lungs scored in AR tadpoles and frogs, 43 or 34% had recovered to a normal length. Of these 43, 84% were fully inflated, 12% were inflated proximally but not at the distal tip, and two were tissue-filled. Of the 85 lungs that were shorter than normal length, 45% appeared to be absent, 15% were fully inflated, 13% were curled, and one was tissue-filled. Of the 62 AR specimens with two scoreable lungs, 87% had two straight lungs, 8% a curled left lung and straight right one, and 5% two curled lungs. Of the 11 specimens with differently sized lungs, 67% had longer right lungs.

### Lung recovery and behaviors of 8-week old AR tadpoles

**Fig. 3.**
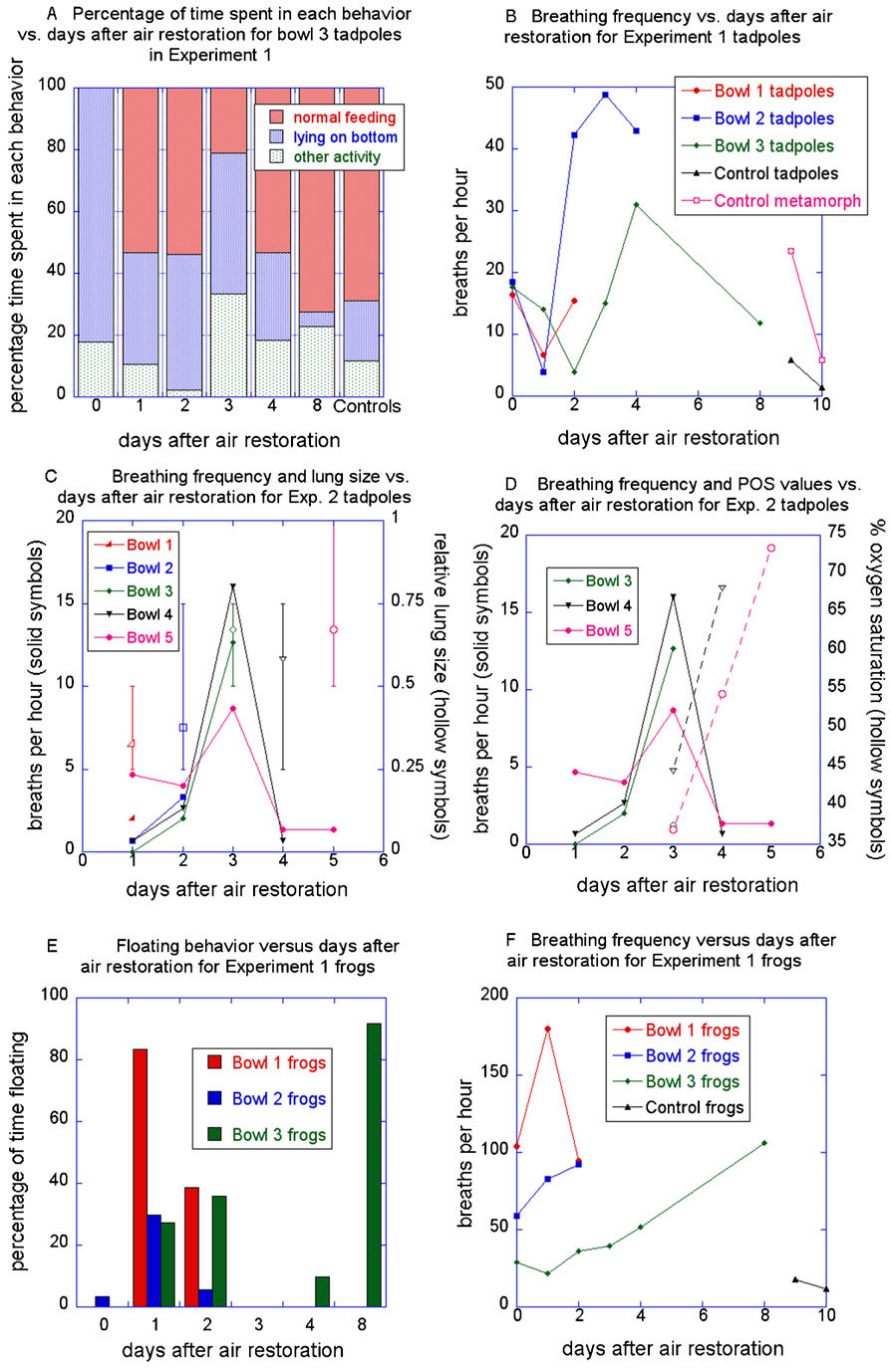
Swimming and breathing behaviors as a function of days after air restoration for experiments 1 and 2 on 8-week old tadpoles and frogs. (A) Swimming behaviors for tadpoles in bowl 3 and controls of experiment 1. (B) Breathing frequencies for tadpoles in bowls 1–3 of experiment 1. (C) Breathing frequencies and relative lung sizes for tadpoles in bowls 1–5 of experiment 2; error bars indicate maximum and minimum lung lengths of specimens sampled on each day. (D) Breathing frequencies and POS values of the water for tadpoles in bowls 3–5 of experiment 2. (E) Floating behavior for frogs in bowls 1–3 of experiment 1; bowl 1 and 2 specimens were sampled or died before day 3; controls (not shown) did not float. (F) Breathing frequencies for frogs in bowls 1–3 and controls of experiment 1.

In Experiment 1, the percentage of time spent in normal feeding increased from zero immediately after transfer to 30–55 on day 1 (Fig. 3A and bowls 1 and 2, data not shown). This percentage declined on days 2–4 and increased to about 70 on day 8, which was similar to controls. Breathing frequency started at 16–18 breaths per hour on day 0, dropped to about 5 (which was similar to controls) on days 1–2, increased to 30–50 on days 2–4 and dropped to about 10 on day 8 (Fig. 3B). The tadpoles sampled on days 4 and 8 had normal length, inflated lungs (the day 2 lungs were lost to sectioning problems). Experiment 2 tadpoles showed a similar trend in breathing frequency, with a dramatic increase on days 2–3, followed by a drop to below the day 1 value by day 4 and subsequent leveling off (Fig. 3C). POS of the water increased steadily over days 3–5 (Fig. 3D), as did the average lung size of tadpoles sampled on days 1–5 (Fig. 3C); the highest percentage of normal length, inflated lungs occurred on day 3. In both experiments, animals engaged in normal feeding tended to position their bodies more vertically on days 1 and 2 relative to later days and untreated tadpoles.

### Lung recovery and behaviors of AR frogs

In Experiment 1, the 8-week old AR frogs did not exhibit consistent trends in floating behavior (Fig. 3E) or breathing frequency (Fig. 3F); frequencies were generally higher than for 8-week old AR tadpoles and for control frogs of comparable sizes. For the one bowl for which day 8 data are available, breaths per hour increased from 25 on day 0 to 100 on day 8, though the latter value is probably misleading as it results from one breath taken during a very short period of submergence. The one frog for which lung size data are available had 0.25 PCL, uninflated lungs on day 8.

Experiment 3 involved five 11-week old frogs transferred to a nonaerated circular tank (depth or D = 6 cm). Immediately after transfer, the frogs stayed submerged and took 18 breaths per hour; a frog sampled after the observation period had no lungs. After week 1 with aeration, three frogs floated continuously and the fourth spent 83% of its time submerged and did not breathe while submerged; one floating frog had 0.5–0.75 PCL, inflated lungs and the mostly submerged frog had 0.25 PCL, uninflated lungs. At week 4, the two remaining frogs continued to float in nonaerated water, but stayed submerged in aerated water and took 20 breaths per hour; these frogs had no lungs.

Experiment 4 involved three 21-week old frogs transferred to a filled fishbowl (D = 13 cm) with aeration, three others transferred to a filled fishbowl without aeration and three similarly sized control frogs transferred to a filled fishbowl without aeration. Immediately after transfer, the AR frogs with and without aeration floated 88 and 97% of the time respectively and did not breathe while submerged. The controls floated 5% of the time and appeared to take 165 breaths per hour, though their continuous activity made it difficult to distinguish between breaths and efforts to escape the bowl. POS values, which had been 99 and 91 for bowls with and without aeration before transfer, dropped to 98 in the aerated bowl with AR frogs, 87 in the nonaerated bowl with AR frogs, and 86 in the nonaerated bowl with control frogs after the 30-minute observation period. The three AR frogs in the nonaerated bowl died less than 24 hours later and had no sign of lungs. On day 1, the surviving AR and control frogs, whose fishbowl POS values had dropped to 93 and 57 respectively, were transferred to aerated rectangular tanks (D = 8 cm). Their breath frequencies were respectively 19 and 25 at week 2 and 25 and 16 at week 4. At 29 days, the AR frogs were moved to a similar tank without aeration, the POS value of which dropped from 97 before transfer to 83 at ten hours and then leveled off. At day 30, these frogs had one normal length, inflated lung and five absent lungs.

Experiment 5 involved three 26-week old frogs and three similarly sized control frogs transferred to nonaerated rectangular tanks (D = 8 cm). On days 0 and 1, the AR frogs floated 21 and 71% of the time and did not breathe while submerged. One AR frog sampled on day 1 had 0.5 and 0.75 PCL, inflated lungs and the other two were missing lungs. The control frogs were too active to measure floating or breathing frequency; older untreated frogs that were acclimated to their tank stayed submerged and breathed 18 and 27 times per hour on two consecutive days.

To summarize these results, with the exception of Experiment 4, AR frogs tended to stay submerged immediately after transfer, but once acclimated to the new tank tended to float in nonaerated water and stay submerged in aerated water. In Experiment 4, all frogs were transferred initially to full fishbowls, the surface area-to-volume ratio (0.04) of which was much lower than for the half-full fishbowls and tanks used in other experiments (0.13–18); the AR frogs that received no aeration died. Lung recovery in all experiments did not correlate with age, aeration or changes in breathing or swimming behavior as the behavior of the few frogs with one or two recovered lungs was not consistently different from that of cohorts with stunted or absent lungs.

## Discussion

### The effects of air deprivation on *Xenopus* biology

In the one previous study to raise *Xenopus laevis* tadpoles in normoxic water without access to air (Pronych and Wassersug, 1994), the negative effects of this treatment on growth rate and lung and heart development were severe enough for the authors to conclude that lung ventilation was obligatory in the tadpoles of this species (Wassersug, 1996). In their study, only one of over 600 AD tadpoles survived to complete metamorphosis and took 16 weeks to do so. In the current study, the five efforts to raise AD specimens produced 61 specimens that completed metamorphosis and 159 that were sampled at earlier stages. Mortality was infrequent and most deaths were attributable to excessive debris build-up inside cages or aeration failure. The fastest growing AD specimens took seven weeks to complete metamorphosis, which was two weeks longer than their fastest uncaged siblings. In a related study on the effects of air deprivation on developmental and growth rates (C.S.R., unpublished data), being caged and air-deprived did not cause consistently higher mortality than being caged and air-exposed. Further, no dissected AD or AR animals exhibited hearts of unusual size or shape compared to controls. Thus, we conclude that lung respiration is not obligatory in *Xenopus laevis* tadpoles or frogs.

The rate and mortality differences between the two studies might be due to differences in rearing conditions and water circulation. Pronych and Wassersug report using filtered, dechlorinated tap water, replacing the mesh coverings of cages to remove detritus, and using densities of 7 tadpoles per liter (Pronych and Wassersug, 1994); they do not report doing water changes over the 16 weeks. Our efforts to produce AD specimens utilized a pH buffered rearing solution, densities of 3–4 tadpoles per liter, and regular water changes. Past experience in our lab has indicated that the buildup of decaying food and feces can reduce the pH of tap water considerably and leads to slow growth and development for *Xenopus* tadpoles (C.S.R., personal observations). Hence, we raise all *Xenopus* tadpoles in our lab in a salt solution buffered to maintain a pH of 7.0–7.4. We also raise untreated siblings in adjacent tanks to check for fast developers reaching NF 58 by 4–5 weeks. A minimum age of 4–5 weeks for NF 58 is consistent with Nieuwkoop and Faber's average age of 6 weeks and Hilken et al.'s minimum age of 5–7 weeks for NF 66 (Nieuwkoop and Faber, 1956; Hilken et al., 1995). We use it as a standard for demonstrating that food, water, light, temperature and specimen quality are satisfactory for our experiments.

### The developmental significance of lung plasticity in *Xenopus* tadpoles

Given the importance of positive buoyancy for feeding and locomotion in *Xenopus* tadpoles and their unlikelihood of being air-deprived in nature, it is not clear whether their lung plasticity would have any ecological significance. However, in addition to defying the general expectation that model organisms do not have plastic development (Bolker, 1995), this property opens up new avenues for researching respiration and lung development. That *Xenopus* can be induced to develop without functional lungs raises the question of what physiological adaptations and developmental plasticity are available to amphibians to compensate for losing lung respiration. Amphibians that have already lost or reduced their lungs often exhibit morphological adaptations that are expected to augment skin respiration. These include thin epithelia, capillaries lying close to the skin surface, a high density of skin capillaries, and folds or papillae to increase skin surface area (Noble, 1925). Anamniotes also exhibit many adaptive responses for coping with hypoxia. These include developing thinner epithelia, denser capillaries, and more and larger gill filaments in *Rana* tadpoles (Burggren and Mwalukoma, 1983); increasing the number and volume of red blood cells in sharks (Chapman and Renshaw, 2009); and increasing the rate of buccal pumping and lactate accumulation in AD *Xenopus* tadoles (Feder and Wassersug, 1984) and the lungless salamander, *Desmognathus* (Gatz and Piiper, 1979; Sheafor et al., 2000). *Desmognathus* can even restore normal ATP and lactate levels while under hypoxia (Gatz and Piiper, 1979).

Whether *Xenopus* tadpoles modify their skin, capillary, or red blood cell development or their metabolic functions in response to the loss of lung respiration remains to be investigated. Excluding the three that died, specimens in this study probably required no compensatory mechanisms as AD specimens likely received sufficient oxygen from normoxic water across their external skin and AR specimens could supplement their skin respiration by engulfing air into the buccopharyngeal cavity. Whereas *Xenopus* remains fully aquatic throughout life, terrestrial frogs are more likely to require compensatory development at tadpole stages to cope without lung respiration later in life.

In addition, that *Xenopus* tadpoles can recover lungs at a predictable, rapid rate provides a new experimental model for investigating the role of air inflation in lung development. Mammal lungs develop embryonically and begin to grow *in utero*, where they secrete liquid that builds up inside the lung when the tracheal cavity is temporarily closed off from the gut tube. The resulting pressure contributes to partial inflation of the lung as well as lung branching and lung cell differentiation before birth (Tschumperlin et al., 2010). After birth, lung growth and remodeling rely upon stretching of the lung wall by air pressure to promote and maintain the required gene expression and cell activities (Boudreault and Tschumperlin, 2010; Gilbert and Rannels, 1998; Rannels, 1989; Tschumperlin et al., 2010; Yang et al., 2000). Though the onset of air breathing at birth coincides with rapid and dramatic increases in lung vasodilation and vascular remodeling (Leffler et al., 1984; Leone and Finer, 2006; Mortola, 2006), the effects of this transformative event are difficult to isolate *in vivo* and impossible to simulate *in vitro*.

This study shows that under our rearing conditions, AD *Xenopus* tadpoles will reliably attain normal sized, inflated lungs within 8 days after being restored to air, with inflation typically occurring during rapid breathing on days 1–3. Inflation is likely driven by buccal pumping and hydrostatic pressure that pushes air from the buccopharyngeal cavity into the trachea, bronchi and lungs as the tadpole descends from the surface. Inflation is marked by the resumption of normal feeding, swimming and breathing frequency, and by an increase in the dissolved oxygen content of tank water as lung respiration begins to lessen the burden on skin respiration. The predictability of lung inflation, its visibility in albino specimens, and its complete inducibility in tissue that otherwise remains unchanged suggest that induced lung inflation in *Xenopus* might be used to identify changes in gene expression, cell differentiation and cell behavior that result from the increased surface tension, stretching, and oxygen pressure that accompany the onset of breathing (Mortola, 2006).

The results of this study show that lung inflation and subsequent breathing do not stimulate cell division in lung tissue and are not necessary for the formation of veins, cartilage and smooth muscle bands in the lung wall. Inflation is necessary for the initial expansion of the lung lumen and thinning of lung walls, and subsequent breathing is necessary for the separation of smooth muscles bands into widely spaced bundles and the subsequent thinning and lengthening of epithelial folds into alveolar septa. Whether the physical forces generated by breathing are also necessary for the differentiation of specific cell types and extracellular fibers remains to be investigated.

### Why do *Xenopus* frogs not recover their lungs?

The low occurrence of lung recovery in AR frogs and its lack of correlation with behavior, age/size and water aeration indicate that lung recovery becomes both improbable and unpredictable at postmetamorphic stages. As shown in Experiment 4, lung recovery also cannot be stimulated in frogs by oxygen-stressing them to the point of death, at least within a 24-hour time frame. Numerous physical changes and developmental abnormalities might be expected to compromise the ability of lungs to inflate after metamorphosis. These include lung buds being compressed by distension of the gut tube or the growth of adjacent fat bodies and liver (Fig. 2E), folded lung walls losing the ability to stretch in response to air pressure by developing a thick connective tissue layer with cartilage and veins (Fig. 2F,J,K), the lung lumen filling with blood cells as a result of abnormal vascularization in folded lung tissue (Fig. 2F,L), the lung bud regressing as a result of blood circulation failing in folded tissue (Fig. 2H,I), and thickening of the throat and body walls that would resist hydrostatic pressure pushing air from the buccopharyngeal cavity into the lungs. Hence, only individuals that experience some amount of lung inflation as an AD tadpole, perhaps by inhaling stray gas bubbles or water in place of air, might be able to inflate their lungs as AR frogs. That right lungs more often recover to longer lengths than left lungs suggests that the stomach does affect lung recovery by distending into unoccupied space in the dorsal left part of the pleuroperitoneal cavity.

Also, except for the youngest AR frogs, which had variable breathing and floating behaviors, AR frogs in nonaerated water spent most or all of their time floating and did not surface to breathe when submerged regardless of their lung state. In contrast, AR frogs in aerated water tended to stay submerged and surface to breathe at frequencies comparable to controls. Thus, in the absence of water aeration, AR frogs appear to require continuous gas exchange with air across the buccopharyngeal epithelium to supplement their skin respiration in water. In the presence of water aeration, skin respiration is apparently sufficient for AR frogs to resume the normal breathing behavior that would be required for lung recovery to occur. The POS measurements indicated that, with the probable exception of Experiment 4, AR frogs were able to respire adequately without the water dropping below normoxic levels. In Experiment 4, the low surface area-to-volume ratio of the filled fishbowl coupled with no aeration presumably resulted in a rate of oxygen supply to the water below what was required for skin respiration to sustain life.

### The evolutionary significance of lung plasticity in amphibians

Lungs occur in tetrapods, lungfish, coelocanths, and three basal actinopterygians, the bichir, alligator gar and bowfin. Given that amphibians are the only tetrapods with multiple organs that play major roles in gas exchange, they are the only tetrapods with the potential to lose their lungs evolutionarily. Indeed, frogs and salamanders have both evolved lung reduction multiple times, all three groups of lissamphibians have evolved lung loss at least once, and its occurrence in plethodontid salamanders is associated with impressively high speciation (Bickford et al., 2008; Duellman and Trueb, 1986; Dunn, 1923; Dunn, 1926; Noble, 1931; Nussbaum and Wilkinson, 1995; Wilkinson and Nussbaum, 1997). One argument for why lung loss evolves is that lungs are no longer needed by animals living in cool, highly oxygenated water so they become eliminated by natural selection to conserve energy (Noble, 1925; Noble, 1931). Another is that they are eliminated by selection to reduce positive buoyancy, which destabilizes larval animals in fast flowing water and allows them to be swept downstream (Beachy and Bruce, 1992; Wilder and Dunn, 1920). Distinguishing between these hypotheses is difficult as habitats with cool, highly oxygenated water, i.e., mountain streams, are often fast flowing. Also, some salamanders with highly reduced lungs, e.g., *Salamandrina terdigitata*, do not live in mountain streams, whereas others with well developed lungs, e.g., *Ranodon sibiricus*, do (Noble, 1929). Bruce et al. have demonstrated that inducing stunted lungs in a normally lunged salamander reduces the tendency of larvae to be swept downstream (Bruce et al., 1994). Beachy and Bruce further argue that lung loss would be most adaptive in fast-water species with large, long-lived aquatic larvae such as ancestral plethodontids (Beachy and Bruce, 1992). Alternatively, *Rhyacotriton*, which has highly reduced lungs and a multiyear larval period and can inhabit cold, but shallow, slow moving water (Czopek, 1962; Welsh and Lind, 1996), might have evolved its lung reduction in response to high oxygen availability. Though cold, deep water might also favor lung reduction, only one amphibian, *Necturus*, is known to occupy this habitat (Reigle, 1967) and its lung development at depth remains unstudied.

In light of renewed appreciation for the importance of developmental plasticity in morphological evolution (Gilbert and Epel, 2009; West-Eberhard, 2003), the findings of this study offer new insight into the roles of lung developmental plasticity in lung loss and life history evolution. The lungless *Xenopus* induced here suggest that lung loss arises from failure to inflate lung buds, which leads to their subsequent regression and disappearance. Indeed, plethodontids exhibit lung buds that never develop a lumen and regress at embryonic stages (Mekeel, 1926; Mekeel, 1930). The current study also shows that timing of lung inflation and later lung development is plastic and controlled by the onset of breathing. The urge to start breathing appears to be genetically fixed in placental mammals by prenatal neurological mechanisms (Champagnat et al., 2011), but occurs gradually enough in birds and other amniotes to suggest that it might be induced by oxygen demand (Mortola, 2006). Since amphibian larvae benefit from being small and having multiple organs for gas exchange, their onset of breathing is likely determined by the effects of lung inflation on other functions such as buoyancy, locomotion and feeding.

Regardless of the underlying mechanism, the variability in onset of lung use that is afforded by lung plasticity has multiple implications for life history evolution. Delaying lung inflation to late larval or metamorphic stages is expected to delay the formation of secondary and tertiary septa to a larger body size after metamorphosis. Species that utilize negative buoyancy as tadpoles and maximal partitioning of lung space as adults, e.g., bufonids (Okada et al., 1962; Smith and Campbell, 1976; Wassersug and Seibert, 1975), might therefore be constrained to have short larval periods and small metamorphs. This constraint would be reinforced by metamorphs moving to land as lung inflation is likely assisted by hydrostatic pressure that helps compress throat and body walls and generate the air pressure required to stretch out lung walls.

Delaying lung inflation until after metamorphosis introduces the risk of lung inflation being compromised by the factors discussed above. The longer the larval period, the greater the risk becomes as body parts become larger and heavier and the animal becomes more reliant upon other specializations for gas exchange and coping with or avoiding oxygen stress (Gatz and Piiper, 1979; Noble, 1929). It is certainly possible to envision an ecological niche that requires no lung inflation in long-lived larvae and lungs with little partitioning in adults. However, lunged species that never develop septa and rely primarily on skin respiration as adults generally inflate their lungs as larvae, e.g., *Triturus* spp. (Czopek, 1965; Goniakowska-Witalińska, 1980; Okada et al., 1962). With the possible exception of *Rhyacotriton* spp. (Czopek, 1962), no amphibians undergo lung inflation after a multiyear larval period, which suggests that a delay of this extent predisposes lung inflation to fail. Lastly, even though all lung functions appear to be lost when lungs fail to inflate, lung buds are not easily lost in evolution. The resemblance of some tissue-filled lungs in this study to spleen tempts the speculation that uninflated lung buds could evolve a new immune function, though to date there is no evidence of this occurring in nature.

## Materials and Methods

### Rearing, sampling, and preparing specimens

Specimens were produced from overnight matings of *Xenopus laevis* injected with 200–500 units of human chorionic gonadotropin (Sigma C1063); females were preinjected with 50–100 units of pregnant mares serum gonadotropin (Sigma G4877) four days previously. Specimens were reared under natural lighting at room temperature (22–24°C) in 0.1× Marc's Modified Ringers (MMR) and fed every 1–2 days with a tadpole chow suspension or sinking frog pellets (*Xenopus* Express). Partial water changes were done every 2–3 weeks. Specimens were staged using the Nieuwkoop Faber (NF) staging system for *Xenopus laevis* (Nieuwkoop and Faber, 1956) and categorized as tadpoles (NF 46–58), metamorphosing specimens or metamorphs (NF 59–65) and postmetamorphs or frogs (≥NF 66) stages.

Untreated specimens were raised in half-filled 1-liter boxes for the first week and half-filled 8-liter tanks thereafter. AD specimens at NF 46 were produced by transferring embryos at 3–5 days postfertilization (pf, NF 41–46) to petri dishes that were filled and lidded to exclude air. AD specimens ≥NF 47 were produced by raising five batches of animals in polypropylene screen cages. For each batch, 30–40 embryos at 3 days 4 hours pf (NF 41–42) were transferred into two cages (13×12×17 cm, V = 2.65 l) that were fully immersed in a 5-gallon tank (24×20×40 cm, V = 19.20 l). The tank was equipped with four air stones for aeration and a pump that circulated water for four 1.5-hour periods every day. The cage screen had an opening size of 0.65 mm and thread diameter of 0.29 mm. Videotape of animals being raised in cages can be viewed in supplementary material Movie 1. Percent oxygen saturation (POS) values for water, which were measured outside the cages using a YSI 556MPS, ranged from 92 to 97. Specimens were removed from cages by inserting a dip net through a closable hole in the plastic roof of each cage. AR tadpoles and metamorphs were transferred to half-filled drum fishbowls (V = 0.65 l, SA = 96 cm^2^) without aeration. Depending on the experiment, AR frogs were transferred to a half-filled or filled drum fishbowl (V = 1.1 l, SA = 45.4 cm^2^) or a circular or rectangular tank (V = 2.3 or 4.2 l, SA = 380 or 532 cm^2^) with or without aeration. All air restoration experiments were done at densities of 2–4 individuals per container to score the swimming and breathing behaviors of individual specimens; tadpoles at these densities did not exhibit schooling behavior (Katz et al., 1981).

Untreated specimens were sampled directly from a tank, AD specimens directly from a petri dish or cage, and AR specimens after varying intervals following transfer to a fishbowl or tank. Some specimens in each category were treated prior to sampling with bromodeoxyuridine (BrdU, Sigma B5002) to analyze cell division in lung tissue. Embryos and tadpoles younger than 1 week pf (≤NF 46) were immersed in 0.3% BrdU in 0.1× MMR for 6, 12, 24 or 72 hours before sampling. Older tadpoles were injected intraperitoneally with 4–6 µl of 0.05% BrdU in deionized water 24 hours before sampling. When handling AD specimens, care was taken to minimize the time spent in air and to keep netted specimens submerged during injection and euthanization. All specimens were euthanized by immersion for 15 minutes in 0.1% MS222 (Sigma A5040).

Specimens were prepared as whole mounts, histological sections or both. Most whole mounted specimens were fixed in 10% neutral-buffered formalin for 48 hours, stored in 70% ethanol, and staged, dissected and photographed under a Zeiss Stemi SV 11 dissecting scope. Some were stained with a Cy3 antibody for α-smooth muscle actin (Sigma C6198) and photographed under a Leica MZ 16 F fluorescent stereomicroscope. Histological specimens were staged, wax-embedded, sectioned at 6 or 10 µm in frontal, transverse and sagittal planes, and stained with hematoxylin and alcian blue and direct red for connective tissues. Some slides were stained with DAPI and the Cy3 antibody for α-smooth muscle actin, and photographed under a Leica DM 2500 fluorescent compound scope. Slides of BrdU-treated specimens were processed with standard immunohistochemical techniques using the G3G4 antibody for BrdU (Developmental Studies Hybridoma Bank), peroxidase-diaminobenzidine labeling, and hematoxylin counterstaining.

To test for long-term effects of air deprivation on frog survivorship and lung development and recovery, 12 AD frogs were transferred to an 11-liter, coarse-mesh cage immersed in a 16-liter tank with aeration at 4 months of age. Water was changed once a week and the cage walls were scrubbed to remove debris when necessary. Seven frogs were removed from the cage at 10–15 months and transferred to normal holding tanks without aeration. One AD and 4 AR frogs were sampled at 19 and 14.5 months respectfully, 2 AR frogs were lost to containment problems at 17 months, and 3 AD and 2 AR frogs have been alive for 24 months at the time of publication.

### Description and scoring of lung development

Lungs were examined in 188 untreated animals, 135 AD animals and 75 AR animals (see supplementary material Table S1 for a breakdown by preparation method and stage). To quantify the effects of air deprivation and restoration on lung development, the unstained lungs of untreated, AD and AR whole mounts at NF≥49 were scored for the following features: length relative to the length of the pleuroperitoneal cavity; anterior or dorsal curling of the posterior tip of the lung; inflation, which was indicated by transparent lung surfaces bulging outwardly from a lattice-like network of opaque partitions that correspond to primary alveolar septa (Burggren and Mwalukoma, 1983; Waterman, 1939) and large lumens that often contained air bubbles (uninflated lungs in contrast appeared as opaque white dense tissue that lacked bulging surfaces, partitions, lumens and air bubbles); and tissue-filling, which was indicated by a large, apparently inflated lumen filled with solid white tissue. Three AD and three AR lungs could not be scored because of damage in dissection; when possible, frontally sectioned AD and AR specimens were also scored to increase sample sizes.

### Assessment of swimming and breathing behaviors in AR specimens

Five experiments were done to assess the rate and degree of lung recovery of AR animals as functions of time after air restoration, stage, age, and aeration and to correlate this process with changes in swimming and breathing behaviors. Experimental designs were varied to pinpoint the timing of lung recovery in tadpoles, to stimulate lung recovery in frogs and to identify behavioral changes that would indicate when it had occurred.

Experiments 1 and 2 assessed lung recovery in 8-week old AR tadpoles and frogs. In Experiment 1, three tadpoles and one frog were transferred from cages to each of three nonaerated, half-filled drum fishbowls and videotaped for 5-minute periods on days 0–4 and 8 after removal from cages; controls of similar age and stage were videotaped on two consecutive days. The specimens in bowls 1, 2 and 3 were respectively BrdU-injected on days 1, 3 and 7, sampled after videotaping on days 2, 4 and 8 and sectioned frontally for immunohistochemistry and scoring lung development. Portions of videotape from Experiment 1 can be viewed in supplementary material Movies 2–15. In Experiment 2, five nonaerated, half-filled drum fishbowls each containing three tadpoles were observed for 30 minutes on days 1–5 after transfer; one bowl of specimens were sampled after each observation period to score lung development. Tank depth for experiments 1 and 2 (8 cm) was similar to depths used in other experiments on *Xenopus* tadpole behaviors (Calich and Wassersug, 2012; Katz et al., 1981).

Experiments 3–5 assessed lung recovery in 11-, 21- and 26-week old AR frogs respectively. In Experiment 3, five frogs were observed in a nonaerated circular tank for 30-minute periods immediately and 7 and 28 days after transfer from cages. In Experiments 4 and 5, nine frogs were filmed for one or more 30-minute periods on days 0, 1, 14 and 28; aeration and tank shape and depth were varied in one or both experiments. Some specimens were sampled after each observation or filming period to score lung development.

AR tadpoles were scored for three categories of swimming behavior. The first, normal feeding, was defined by the longitudinal axis of the body being 30–80 degrees from the horizontal (head end down), at rest except for tail beating and feeding/ventilation movements of the head (buccal pumping), not sinking or rising but possibly drifting (meaning a slow forward movement with or without changing depth), and either in the water column or just above the bottom; this category accords with Gradwell's description of phasic water pumping (Gradwell, 1971). The second, lying on the bottom, was defined by the body being 0–30 degrees from the bottom and the belly touching the bottom; it sometimes included buccal pumping. The third, other activity, included swimming upwards or downwards, floating or swimming horizontally back and forth at or near the surface, floating horizontally in the water column, and sinking; this category included Gradwell's searching behavior and did not include buccal pumping (Gradwell, 1971). These behaviors were quantified by using Macintosh IMovie 11 to divide videotape sequences into 10-second clips and scoring the predominant category of behavior for each tadpole in each clip. The scores for each category were then totaled and normalized for observation time and number of tadpoles. AR tadpoles were also scored for frequency of breaths, which were recognized as surfacing followed by rapid submerging and the release of bubbles less than 3 seconds later.

AR frogs were scored for breathing frequency and percentage of time spent floating. Breaths were recognized as rapidly protruding the snout above water before submerging, a series of rapid snout protrusions lasting less than 30 seconds, and floating with the snout above water for less than 30 seconds. Frog floating and submerged times were tallied from timed observations or 30 second clips of videotape, and only periods of continuous floating that lasted longer than 30 seconds were counted. Breathing frequencies were calculated using the total length of submerged time.

## Supplementary Material

Supplementary Material
