# Supplementary material for: Plasticity of lung development in the amphibian, Xenopus laevis
Source: Biol Open. 2013 Oct 16;2(12):1324–35. doi: 10.1242/bio.20133772 (PMC3863417; doi:10.1242/bio.20133772)
Supplement: Supplementary Material [file supp_2_12_1324__index.html]

Plasticity of lung development in the amphibian, Xenopus laevis — Supplementary Material 

# Plasticity of lung development in the amphibian, *Xenopus laevis*

## bio.20133772 Supplementary Material

**Files in this Data Supplement:**

- Supplementary Material - Christopher S. Rose and Brandon James doi: 10.1242/bio.20133772
- Movie 1 - **Movie 1. A 10-second clip of air-deprived *Xenopus* tadpoles, metamorphs and frogs that have been raised in a cage for 8 weeks. The aeration has been turned off to eliminate bubbles.**
- Movie 2 - **Movie 2. A 10-second clip of untreated (control) tadpoles and metamorphs in Experiment 1 showing swimming and breathing behaviors.**
- Movie 3 - **Movie 3. A second 10-second clip of untreated (control) tadpoles and metamorphs in Experiment 1 showing swimming and breathing behaviors.**
- Movie 4 - **Movie 4. A 10-second clip of tadpoles and frog in Experiment 1 Bowl 3 that have been air-restored (AR) for 0 days, showing swimming and breathing behaviors.**
- Movie 5 - **Movie 5. A second 10-second clip of tadpoles and frog in Experiment 1 Bowl 3 that have been air-restored (AR) for 0 days, showing swimming and breathing behaviors.**
- Movie 6 - **Movie 6. A 10-second clip of tadpoles and frog in Experiment 1 Bowl 3 that have been air-restored (AR) for 1 day, showing swimming and breathing behaviors.**
- Movie 7 - **Movie 7. A second 10-second clip of tadpoles and frog in Experiment 1 Bowl 3 that have been air-restored (AR) for 1 day, showing swimming and breathing behaviors.**
- Movie 8 - **Movie 8. A 10-second clip of tadpoles and frog in Experiment 1 Bowl 3 that have been air-restored (AR) for 2 days, showing swimming and breathing behaviors.**
- Movie 9 - **Movie 9. A second 10-second clip of tadpoles and frog in Experiment 1 Bowl 3 that have been air-restored (AR) for 2 days, showing swimming and breathing behaviors.**
- Movie 10 - **Movie 10. A 10-second clip of tadpoles and frog in Experiment 1 Bowl 3 that have been air-restored (AR) for 3 days, showing swimming and breathing behaviors.**
- Movie 11 - **Movie 11. A second 10-second clip of tadpoles and frog in Experiment 1 Bowl 3 that have been air-restored (AR) for 3 days, showing swimming and breathing behaviors.**
- Movie 12 - **Movie 12. A 10-second clip of tadpoles and frog in Experiment 1 Bowl 3 that have been air-restored (AR) for 4 days, showing swimming and breathing behaviors.**
- Movie 13 - **Movie 13. A second 10-second clip of tadpoles and frog in Experiment 1 Bowl 3 that have been air-restored (AR) for 4 days, showing swimming and breathing behaviors.**
- Movie 14 - **Movie 14. A 10-second clip of tadpoles and frog in Experiment 1 Bowl 3 that have been air-restored (AR) for 8 days, showing swimming and breathing behaviors.**
- Movie 15 - **Movie 15. A second 10-second clip of tadpoles and frog in Experiment 1 Bowl 3 that have been air-restored (AR) for 8 days, showing swimming and breathing behaviors.**
