# Supplementary material for: Plasticity of lung development in the amphibian, Xenopus laevis
Source: Biol Open. 2013 Oct 16;2(12):1324–35. doi: 10.1242/bio.20133772 (PMC3863417; doi:10.1242/bio.20133772)
Supplement: Supplementary Material [file supp_bio.20133772_bio.20133772-s1.pdf]

Supplementary Material

Christopher S. Rose and Brandon James **doi: 10.1242/bio.20133772**

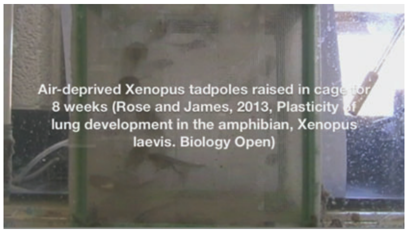

Movie 1. A 10-second clip of air-deprived *Xenopus* tadpoles, metamorphs and frogs that have been raised in a cage for 8 weeks. The aeration has been turned off to eliminate bubbles.

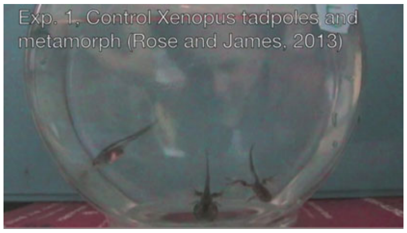

Movie 2. A 10-second clip of untreated (control) tadpoles and metamorphs in Experiment 1 showing swimming and breathing behaviors.

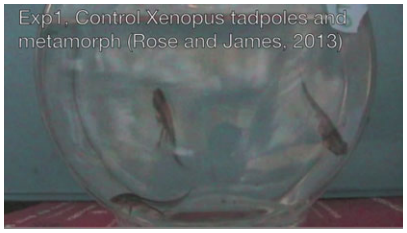

Movie 3. A second 10-second clip of untreated (control) tadpoles and metamorphs in Experiment 1 showing swimming and breathing behaviors.

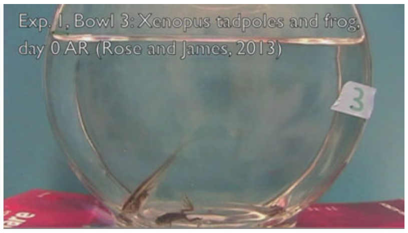

Movie 4. A 10-second clip of tadpoles and frog in Experiment 1 Bowl 3 that have been air-restored (AR) for 0 days, showing swimming and breathing behaviors.

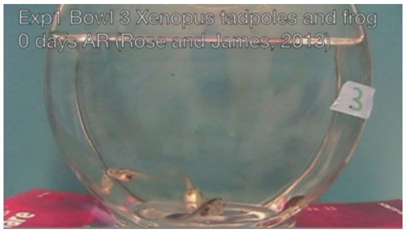

Movie 5. A second 10-second clip of tadpoles and frog in Experiment 1 Bowl 3 that have been air-restored (AR) for 0 days, showing swimming and breathing behaviors.

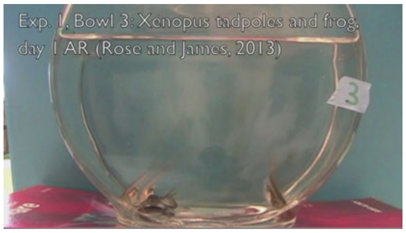

Movie 6. A 10-second clip of tadpoles and frog in Experiment 1 Bowl 3 that have been air-restored (AR) for 1 day, showing swimming and breathing behaviors.

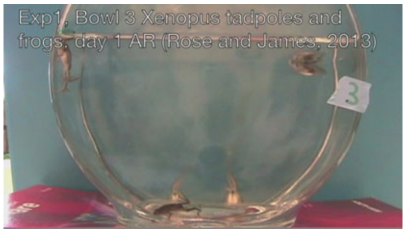

Movie 7. A second 10-second clip of tadpoles and frog in Experiment 1 Bowl 3 that have been air-restored (AR) for 1 day, showing swimming and breathing behaviors.

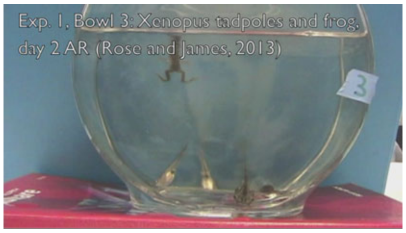

Movie 8. A 10-second clip of tadpoles and frog in Experiment 1 Bowl 3 that have been air-restored (AR) for 2 days, showing swimming and breathing behaviors.

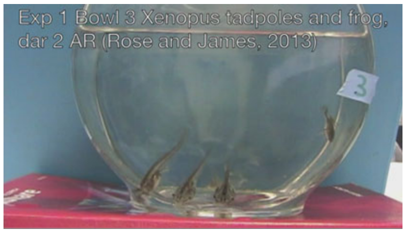

Movie 9. A second 10-second clip of tadpoles and frog in Experiment 1 Bowl 3 that have been air-restored (AR) for 2 days, showing swimming and breathing behaviors.

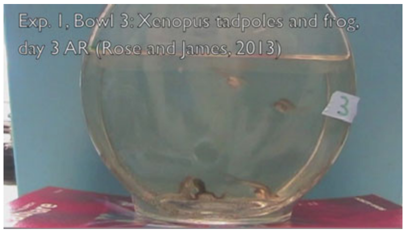

Movie 10. A 10-second clip of tadpoles and frog in Experiment 1 Bowl 3 that have been air-restored (AR) for 3 days, showing swimming and breathing behaviors.

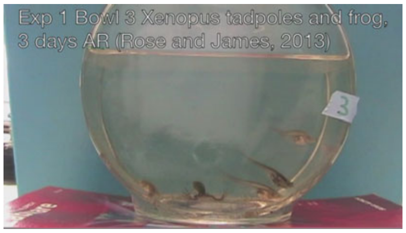

Movie 11. A second 10-second clip of tadpoles and frog in Experiment 1 Bowl 3 that have been air-restored (AR) for 3 days, showing swimming and breathing behaviors.

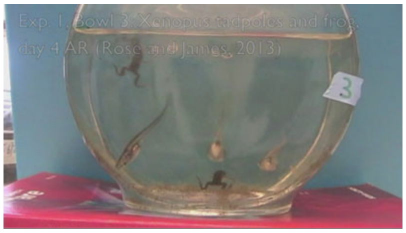

Movie 12. A 10-second clip of tadpoles and frog in Experiment 1 Bowl 3 that have been air-restored (AR) for 4 days, showing swimming and breathing behaviors.

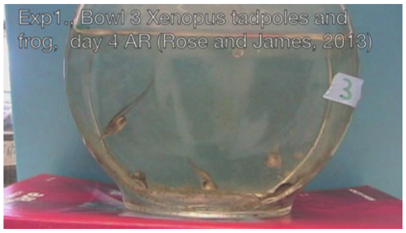

Movie 13. A second 10-second clip of tadpoles and frog in Experiment 1 Bowl 3 that have been air-restored (AR) for 4 days, showing swimming and breathing behaviors.

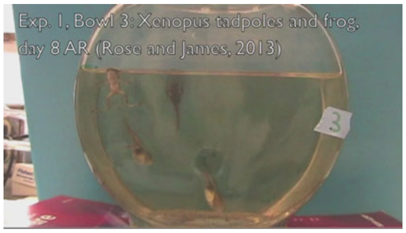

Movie 14. A 10-second clip of tadpoles and frog in Experiment 1 Bowl 3 that have been air-restored (AR) for 8 days, showing swimming and breathing behaviors.

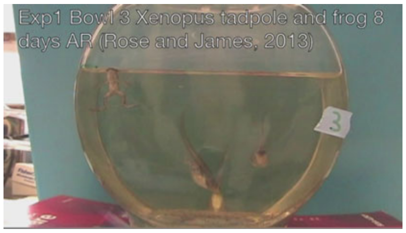

Movie 15. A second 10-second clip of tadpoles and frog in Experiment 1 Bowl 3 that have been air-restored (AR) for 8 days, showing swimming and breathing behaviors.

Table S1. Numbers, stages and/or ages of specimens used for description and scoring of lung development.

	tadpoles	metamorphs	frogs	days reared after air restoration
Untreated specimens:				
Dissected whole mounts	33, NF 51–57		26, NF 66 – 4 months post NF 66	
Albino whole mounts (lungs observed through the skin)	6, NF 46–58	16, NF 60–65	18, NF 66 – 1 month post NF 66	
Smooth muscle actin-stained whole mounts	6, NF 47–56	3, NF 59–65		
Sectioned specimens	73, NF 40–58	10, NF 59–65	7, NF 66 – 6 months post NF 66, fully grown adult	
	(BrdU-treated specimens: 43 at NF 40–47 (11 d pf) and 16 at NF 49–66+; smooth muscle actin-stained specimens: 14 at NF 49–adult.)			
Air-deprived specimens:				
Dissected whole mounts	69, NF 49–57	24, NF 61–65	14, NF 66 – 4 months post NF 66	
Smooth muscle actin-stained whole mounts	3, NF 51–54			
Sectioned specimens	12 at NF 46 (5 days 5 hr pf)*, 9 at NF 49–51 (17 days pf), 4 at NF 56–57	2, NF 64–64.5	2, NF 66, adult at 19 months	
	(*4 NF 46 specimens were made AD at each of NF 41–44 (3 days 16 hours pf), NF 43–45 (4 days 4 hr pf) and NF 46 (4 days 14 hours pf); the first two ages were before the onset of breathing and the third one after. BrdU-treated specimens: 1 at NF 57; smooth muscle actin-stained specimens: 4 dissected whole mount specimens at NF 56, 57, 64 and 64.5 and adult.)			
Air-restored specimens:				
Dissected whole mounts	17, NF 55–58	5, NF 60–65	29, NF 66 – 3 months post NF 66	1–56
Smooth muscle actin-stained whole mounts	3, NF 51–54.5	5, NF 59–64.5		3–9
Sectioned specimens	9, NF 55 and 57		9, NF 66 – 4 months post NF 66, adult at 14.5 months	1–30
	(BrdU-treated: all; smooth muscle actin-stained specimens: 2 dissected whole mounts with tissue-filled lungs at 4 months post NF 66 and adult.)			
